# Clinical Characteristics, Treatments, and Prognosis of Atopic Eczema in the Elderly

**DOI:** 10.3390/jcm4050979

**Published:** 2015-05-18

**Authors:** Ryoji Tanei

**Affiliations:** Departments of Dermatology, Tokyo Metropolitan Geriatric Hospital and Institute of Gerontology, 35-2 Sakaecho, Itabashi, Tokyo 173-0015, Japan; E-Mail: rtanei@aol.com; Tel.: +81-3-3964-1141; Fax: +81-3-3964-1982

**Keywords:** atopic eczema, clinical characteristics, complications, elderly, immunoglobulin E, onset, outcomes, prognosis, skin manifestations, treatment

## Abstract

Atopic eczema (AE) in the elderly is gradually increasing and has been added to the classification of AE in recent years. This investigation retrospectively analyzed 60 patients with elderly AE. Among the clinical characteristics, a male predominance, existence of several patterns of onset and clinical course, and associations with immunoglobulin (Ig)E-allergic-status and asthmatic complication were observed. The highest positive-rate and positive-score for serum-specific IgE against *Dermatophagoides farinae* were 83.8% and 2.65 in patients with IgE-allergic AE, and a lower incidence of lichenified eczema in the elbow and knee folds were observed. In terms of treatments and outcomes, clinical improvement and clinical remission were observed in 80.8% and 36.5% of cases, respectively, using standard treatments and combined therapy with oral corticosteroid in severe cases. As for complications and final prognosis, most elderly AE patients reached the end of life with AE, but patients with IgE-allergic AE showed significantly lower incidences of complications of malignancy and death from malignancy. These results indicate that AE in the elderly represents a new subgroup of AE with specific features.

## 1. Introduction

Atopic eczema (AE, also known as atopic dermatitis) is a chronic, relapsing, severely pruritic eczematous dermatitis, characterized by a pathogenesis involving allergic inflammation and skin barrier defects. Prior to the 1960–70s, AE was considered a pediatric disease with good prognosis—known as classic childhood AE—as the onset of AE mostly appeared in the infantile or childhood phase and resolution of AE occurred by outgrowth by puberty in typical cases. From the 1980s, cases of AE either persisting or first appearing in adolescence and adulthood increased, and were termed adult AE. Furthermore, cases of AE manifesting in the senile phase, *i.e.*, elderly AE, have been gradually increasing in industrialized countries recently, in association with the aging of society [1,2]. In recent papers [3,4,5], clinical and histopathological features of elderly AE have been reported and characterized as follows: both immunoglobulin (Ig)E-mediated allergic and non-IgE-allergic forms exist, and the most frequent environmental allergens involved in the IgE-mediated allergic form are house dust mites (e.g., *Dermatophagoides* species), followed by pollens and foods; three main patterns of onset (senile onset, recurrence with a history of classic child AE and recurrence and/or continuation of adult AE) are present; skin manifestations in elderly AE basically match those of adult AE, but the reverse sign of lichenified eczema around unaffected folds of the elbows and knees is more common than the classic sign of localized lichenification in those folds; and IgE+ mast cells and IgE+CD11c+ dermal dendritic cells predominate among the inflammatory cell infiltration of chronic skin lesions in the IgE-mediated allergic form of elderly AE. As a result, a new subgroup of elderly AE has been added to the classification of AE, and AE is therefore now divided into four phases: infantile (age < 2 years), childhood (age 2–12 years), adolescent/adult (age > 12 but <60 years), and elderly (age ≥ 60 years) in a recent edition of a textbook on dermatology [6].

Although the clinical features of elderly AE have been largely characterized, some issues remain unclear in this subgroup: clinical characteristics and their relationships to onset, allergic/non-allergic diathesis, associated allergic diseases and lifetime clinical course of the patient; consensus establishment of suitable treatment procedures; coexisting/underling medical complications; and prognosis and final outcomes.

This study undertook several retrospective analyses of elderly patients with AE in order to address these issues.

## 2. Materials and Methods

### 2.1. Subjects

Sixty AE patients (41 men, 19 women; ≥60 years old; mean age 77 ± 8.6 years) who had been admitted to the Department of Dermatology at Tokyo Metropolitan Geriatric Hospital and the Institute of Gerontology between January 2000 and December 2014 were retrospectively analyzed by reviewing medical records.

All patients fulfilled the diagnostic criteria of Hanifin and Rajka [7]. All patients were examined for serum levels of total IgE and allergen-specific IgE antibodies. According to the serological atopic diathesis, patients were categorized into three groups: IgE-allergic type; indeterminate-allergic type; and non-IgE allergic type. Patients who showed both higher levels of serum total IgE (>400 IU/L) and positive specific IgE antibodies to common environmental allergens were classified as having IgE-allergic AE. Patients who showed normal levels of serum total IgE (≤400 IU/L) and no detectable positive responses of specific IgE to allergens were classified as having non-IgE-allergic AE. In addition, patients who showed positive specific IgE responses with a total IgE level ≤400 IU/L or negative specific IgE responses with a total IgE level >400 IU/L were classified as having indeterminate-allergic AE [8]. This study was approved by the Ethics Committee of the Tokyo Metropolitan Geriatric Hospital and Institute of Gerontology (No. 260301). 

### 2.2. Serum IgE Antibody Levels and Specific IgE-Scoring

Serum total IgE levels (normal ≤400 IU/L) were determined by enzyme immunoassay. Serum levels of specific IgE against common environmental allergens were detected using the multiple antigens simultaneous test (MAST)-26 (SRL, Tokyo, Japan) and revised MAST-33 version (BML, Tokyo, Japan). Results were judged as positive in class 1 and over for MAST-26 and in class 2 and over for MAST-33. The adjusted class intensity (score) [9] of specific IgEs for MAST-26 and MAST-33 were decided as follows: score 1 (mild) = class 1 (4.41–11.2 lumi count) for MAST26 or class 2 (2.78–13.4 lumi count) for MAST33, score 2 (moderate) = class 2 (11.3–20.0 lumi count) for MAST26 or class 3 (13.5–58.0 lumi count) for MAST33, score 3 (strong) = class 3 (20.1–99.9 lumi count) for MAST26 or class 4 (58.1–119 lumi count), 5 (120–159 lumi count) and 6 (160–200 lumi count) for MAST33, and score 4 (very strong) = over class 3 for MAST26 or over class 6 for MAST33. Total and mean class intensity (score) for 26 common allergens for MAST-26 or MAST-33 were calculated as degrees of specific IgEs-sensitization in those patients; the 26 common allergens were as follows: *Aspergillus fumigatus*, *Alternaria tenuis*, beef, *Candida albicans*, cheddar cheese, cat dander, *Cladosporium herbarum*, chicken meat, crab, dog dander, *Dermatophagoides farinae* (*Der f*), egg white, house dust, Japanese cedar, milk, mugwort, *Penicillum notatum*, rice, ragweed mixture, salmon, shrimp, soybeans, sweet vernal grass, Timothy grass, tuna, and wheat pollen.

### 2.3. Clinical Data Collection and Evaluation

Evaluated clinical findings in each groups were as follows: age and gender; age at onset of AE and clinical course; levels of serum total IgE and specific IgEs; peripheral blood eosinophil count; personal history of AE-associated allergic and non-allergic diseases; family history of AE and associated allergic diseases; clinical course of AE; severity of AE; skin manifestations and distributions; treatments and outcomes; medical complications; and prognosis and final outcomes of patients. Each item was collected from the medical records in our hospital and the answers of patients on the basis of their memories to specific questions asked by the dermatologists. Several comparative analyses were conducted combining those multiple items. Severity of AE was scored at the time of severe illness in each patient using the Objective Severity Scoring of Atopic Dermatitis (objective SCORAD; maximum, 83) index [10]. Blood examination in all except for one patient with IgE-allergic AE had been conducted under therapy without oral corticosteroid or cyclosporine. The blood test in the patient with IgE-allergic AE had been performed while the patient was under treatment for bronchial asthma (BA) with oral prednisolone at 10 mg/day. Serum-specific IgE levels in another patient with IgE-allergic AE were not examined because of the medical plan determined for the patient. In the analyses of treatments and outcomes, cases in which the treatment of AE had been carried out for a duration of six months or more were analyzed. The evaluation was as follows: clinical remission: disappearance of skin lesions in more than 95% of lesional areas observed after six months or more only with standard treatments; clinical improvement: disappearance of skin lesions in more than 95% of lesional areas observed within five months with only standard treatments, or after six months or more with standard treatment and oral corticosteroid therapy at a dosage of ≤5 mg/day (daily or occasional use); and refractory: no clinical improvement or clinical remission observed.

In the analyses of complications and final prognosis, the coexisting/underlying disorders and complications of malignancy and death of elderly AE patients were analyzed from personal histories and episodes in our hospital until the end of 2014 using medical records. As a control, 72 patients with benign epidermal cyst (47 men, 25 women; ≥60 years old; mean age, 74.7 ± 7.0 years) who had been treated in our department between January 2000 and December 2014 were also retrospectively analyzed by reviewing medical records. Mean (±SD) follow-up duration from the day of diagnosis of skin disease was 51.3 ± 48.9 months in elderly AE patients and 57.7 ± 46.7 months in control subjects.

### 2.4. Statistical Analysis

All quantitative data are expressed as means ± SD. For quantitative data, a Mann-Whitney U test or Kruskal-Wallis test was used. Differences among qualitative results were compared using Fisher’s exact test. Values of *p* < 0.05 were considered statistically significant. Data analysis was performed using EZR version X software (Saitama Medical Center Jichi Medical University, Saitama, Japan) [11].

## 3. Results

### 3.1. Clinical Characteristics

Clinical and laboratory data in the 60 elderly patients with AE are summarized in Table 1. According to the serological atopic diathesis, these cases were classified into three types as IgE-allergic AE (*n* = 38), indeterminate -allergic AE (*n* = 9), and non-IgE-allergic AE (*n* = 13), and we analyzed each characteristic. The male/female ratio was 41/19, and male predominance was seen in each type. Needless to say, the average of total IgE values of IgE-allergic AE showed a higher value with the significant difference than those of the other two types. However, the significant difference was not observed in the averages of peripheral blood eosinophils count and the Objective SCORAD score, which is a parameter of a clinical severity of AE [10] in these three types. In regard to the personal history of associated diseases of AE, a significant difference was not observed in the incidence of BA, allergic rhino-conjunctivitis (AR), and urticaria. However, it became apparent that the incidence of ichthyosis is significantly lower in IgE-allergic AE as compared with indeterminate-allergic AE and non-IgE-allergic AE. There was no significant difference in family history in each type of elderly AE. 

**Table 1:** Clinical and laboratory data of the 60 elderly patients with atopic eczema (AE).

	IgE-Allergic AE	Indeterminate-Allergic AE	Non-IgE-allergic AE	Total
*n* (%)	38 (63.3)	9 (15.0)	13 (21.7)	60 (100)
Gender, male/female	27/11 **	7/2 **	7/6	41/19 **
Age (years) ¶	76.2 ± 8.7	79.8 ± 7.4	77.9 ± 9.4	77.1 ± 8.6
Total IgE (IU/mL) ¶	6975.3 ± 9908.4 *†	339.4 ± 515.3	87.3 ± 96.0	4487.6 ± 8513.7
Eosinophils (μL) ¶	1001.2 ± 1232.8	423.8 ± 189.2	476.9 ± 374.7	801.0 ± 1028.2
Objective SCORAD¶	38.1 ± 17.4	23.3 ± 7.6	27.2 ± 16.2	33.5 ± 17.0
Associated diseases				
Personal history				
BA	14 (36.8)	4 (44.4)	2 (15.4)	20 (33.3)
AR	20 (52.6)	4 (44.4)	5 (38.5)	27 (45.0)
BA + AR	5 (13.2)	2 (22.2)	0 (-)	7 (11.7)
Urticaria	8 (21.1)	2 (22.2)	2 (15.4)	12 (20.0)
Ichthyosis	1 (2.6) *†	3 (33.3)	3 (23.1)	7 (11.7)
Family history				
Nonexistent	19 (50.0)	3 (33.3)	6 (46.2)	28 (46.7)
BA	6 (15.8)	1 (11.1)	3 (23.1)	10 (16.7)
AR	6 (15.8)	0 (-)	1 (7.7)	7 (11.7)
BA + AR	2 (5.3)	0 (-)	0 (-)	2 (3.3)
AE	10 (26.3)	2 (22.2)	5 (38.5)	17 (28.3)
AE + BA	4 (10.5)	1 (11.1)	2 (15.4)	7 (11.7)
AE + BA + AR	2 (5.3)	0 (-)	0 (-)	2 (3.3)

Abbreviations: AE, atopic eczema; AR, allergic rhino-conjunctivitis (including pollinosis); BA, bronchial asthma; Eosinophils, peripheral blood eosinophil count; Objective SCORAD, Severity Scoring of Atopic Dermatitis (maximum, 83). ¶ Data are given as mean ± standard deviation. * *p* < 0.05, compared with indeterminate-allergic AE; † *p* < 0.05, compared with non-IgE-allergic AE; ** *p* < 0.05, compared male with female.

**Figure 1:**
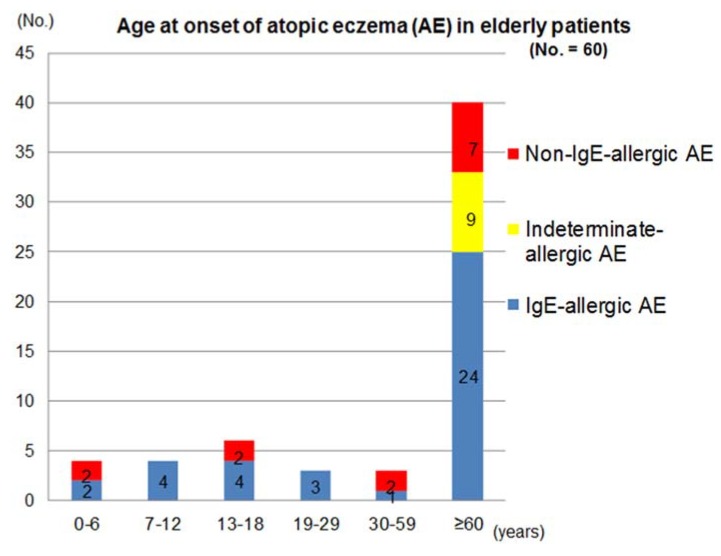
Age at onset of atopic eczema in elderly patients.

The distribution of age of AE-onset and the patterns of clinical courses after the onset in these patients are shown in Figure 1 and Table 2. Two thirds of the patients had senile onset of elderly AE. Since it was based upon the patient’s memory, a patient who reported an onset of AE in an infantile phase (age < 2 years) was the only one in the non-IgE-allergic AE. Onset of AE was seen in the phase of early childhood (0–6 years), late childhood (7–12 years), adolescence (13–18 years), young adulthood (19–29 years), late adulthood (30–59 years), and senile (≥60 years) in the IgE-allergic AE patients; and in the phase of early childhood, adolescence, late adulthood, and senile in the non-IgE-allergic AE patients. On the other hand, the onset of AE was significantly concentrated in the senile phase in the indeterminate-allergic AE patients. The incidence of AE in the elderly patients with IgE-allergic AE and non-IgE-allergic AE in the childhood phase (0–12 years), adolescence/young adulthood phase (13–29 years), late adulthood phase, and senile phase were 15.8% and 15.4%, 18.4% and 15.4%, 2.6% and 15.4%, and 63.2% and 53.8%, respectively. In the non-IgE-allergic AE, although the statistical-significance difference was not obtained, onsets of female patients were predominant in the senile phase. In the IgE-allergic AE and the non-IgE-allergic AE cases, two kinds of clinical course, *i.e.*, senile recurrence type with a history of outgrowing (outgrow-recurrence type) and continuous type were observed both in the cases with childhood onset and adolescence/young adulthood onset.

**Table 2:** Age at onset and clinical course in elderly patients with atopic eczema (AE).

	IgE-Allergic AE	Indeterminate-Allergic AE	Non-IgE-Allergic AE	Total
No. of patients (Male/Female)	38 (27/11)	9 (7/2)	13 (7/6)	60 (41/19)
Onset and course;				
0–12 years				
outgrow and recurrence	3 (2/1)	0 (-)	1 (1/0)	4 (3/1)
continuous	3 (2/1)	0 (-)	1 (1/0)	4 (3/1)
13–29 years				
outgrow and recurrence	2 (2/0)	0 (-)	1 (0/1)	3 (2/1)
continuous	5 (3/2)	0 (-)	1 (1/0)	6 (4/2)
30–59 years *	1 (1/0)	0 (-)	2 (2/0)	3 (3/0)
≥60 years †	24 (17/7)	9 (7/2)	7 (2/5)	40 (26/14)

*,† Basically continuous.

**Figure 2:**
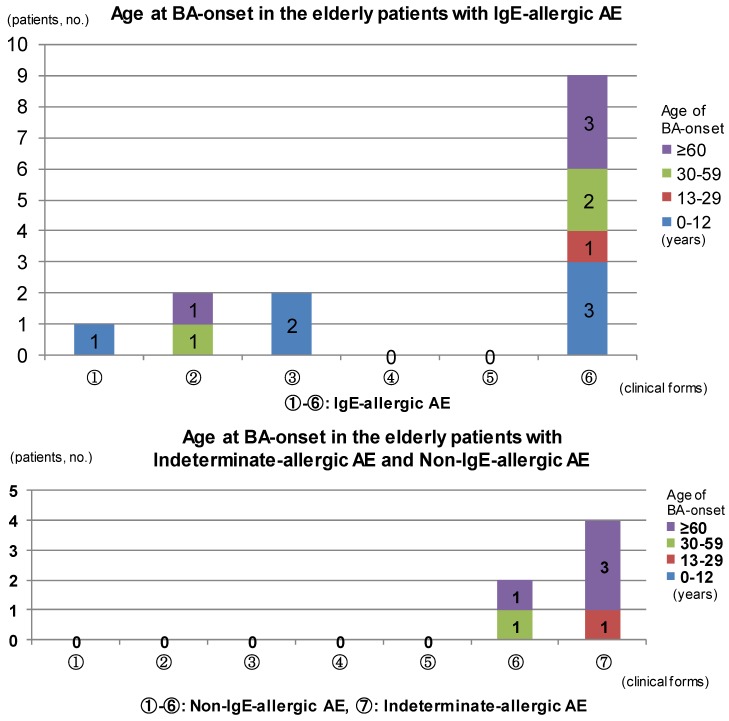
Age at bronchial asthma (BA) onset in elderly patients with atopic eczema. Abbreviations: AE, atopic eczema; BA, bronchial asthma; ① childhood-onset AE, outgrow-recurrence type; ② childhood-onset AE, continuous type; ③ adolescent and young adult-onset AE, outgrow-recurrence type; ④ adolescent and young adult-onset AE, continuous type; ⑤ late adult-onset AE; ⑥ senile-onset AE; ⑦ senile-onset AE.

The results of analysis for complications of BA are summarized in Figure 2. In IgE-allergic elderly AE patients, only childhood BA was seen in the outgrow-recurrence types of childhood-onset AE and adolescent/young adulthood-onset AE as an asthmatic complication. The complications of late adulthood-onset BA and senile-onset BA were seen in the continuous-type childhood-onset AE. Asthmatic complications were not recognized in continuous-type adolescent/young adulthood-onset AE. In senile-onset AE, complications of BA were observed in every phase, *i.e.*, childhood, adolescence/young adulthood, late adulthood, and senile phases (Figure 2, upper bar chart). With indeterminate-allergic AE and non-IgE-allergic AE patients, no personal history of childhood BA was observed and asthmatic complications in which senile-onset BA predominated were recognized only in senile-onset AE (Figure 2, lower bar chart). In cases of IgE-allergic and indeterminate-allergic AE, all asthmatic-merger patients except for two patients with IgE-allergic AE and a personal history of childhood BA showed positive results for serum-specific IgEs against *Dermatophagoides farinae* (*Der f*) In regard to the association between asthmatic complications and total IgE values, the mean total IgE values of elderly AE with a personal history of BA tended to be lower than those of elderly AE without a complication of BA, both in patients with IgE-allergic AE (4917.8 ± 6242.0 IU/mL *vs.* 8175.6 ± 11481.9 IU/mL) and indeterminate-allergic AE (72.8 ± 62.5 IU/mL *vs.* 552.8 ± 632.6 IU/mL), although no significant difference was identified. Complications of RA were recognized in adolescence, adulthood, or senile phases of the patients with elderly AE. As with BA, onset of AR was seen either before, simultaneous to, or after AE onset, but elderly AE patients tended to show an unclear memory of AR-onset as compared with BA-onset.

**Figure 3:**
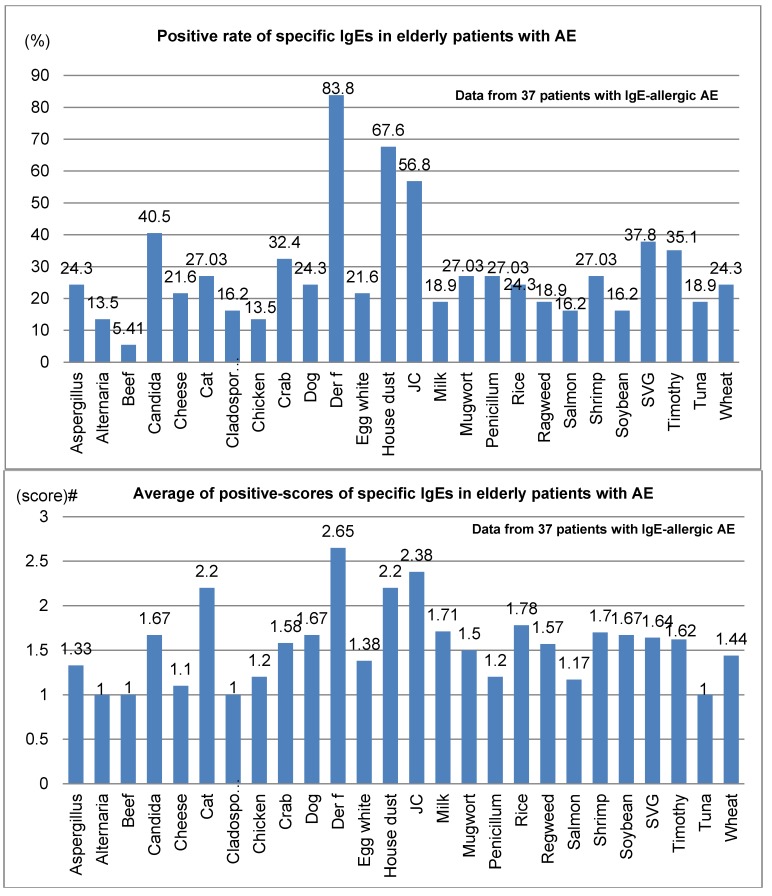
Positive rate (**top**) and average of positive scores (**bottom**) of specific IgEs in elderly patients with atopic eczema. Abbreviations: AE, atopic eczema; Der f, *Dermatophagoides farinae*; JC, Japanese cedar; SVG, sweet vernal grass. # Average of class intensity (score) of the positive allergens for multiple antigen simulations test (MAST)-26 or MAST-33; the adjust class intensity for the common 26 allergens are decided as follows: score 1 (mild) = class 1 for MAST26 or class 2 for MAST33; score 2 (moderate) = class 2 for MAST26 or class3 for MAST33; score 3 (strong) = class 3 for MAST26 or class 4, 5 and 6 for MAST33; score 4 (very strong) = over class 3 for MAST26 or over class 6 for MAST33.

Positive rates and average positive scores for serum-specific IgE for 26 common environmental allergens among 37 elderly patients with IgE-allergic AE are shown in Figure 3. In the positive rates of serum-specific IgE, the positive rate for *Der f* peaked at 83.8%, compared to 67.6% for house dust and 56.8% for Japanese cedar (Figure 3; upper bar chart). Also, the mean positive scores for serum-specific IgE were highest for *Der f*, at 2.65, followed by Japanese cedar, 2.38; house dust, 2.2; and cat dander, 2.2 (lower bar chart, Figure 3). Besides the 26 common allergens, positive rates and mean positive scores for serum-specific IgEs for notable allergens in 18 elderly patients with IgE-allergic AE were as follows: birch, 22.2% and 1.3; peanuts, 16.7% and 1.7; and latex, 11.1% and 1.5, respectively.

**Figure 4:**
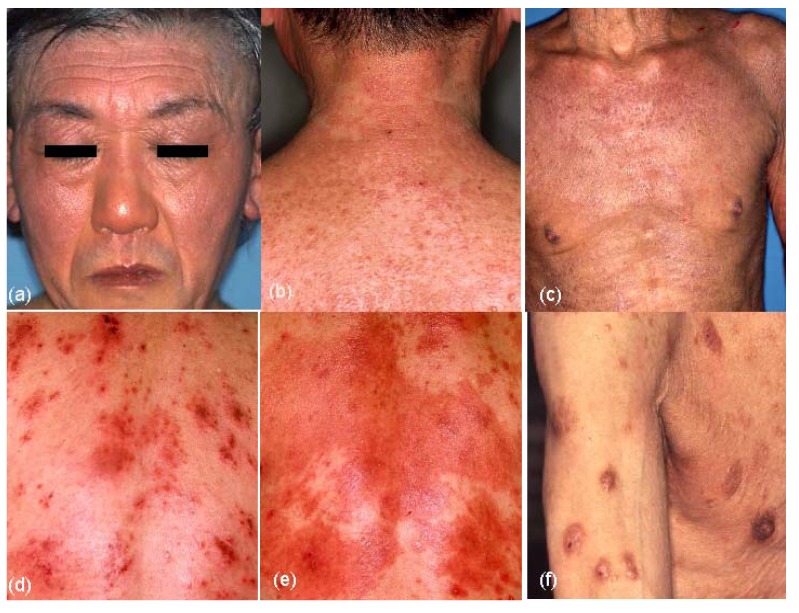
Skin manifestations of elderly patients with atopic eczema (AE). (**a**) Facial eczematous erythema (atopic red face) with Hertoghe’s sign (loss of lateral eyebrows); (**b**) Diffuse eczematous erythema and papules on the neck and upper back; (**c**) Lichenified eczema of erythroderma on the trunk; (**d**) Nummular-form eczema with acute inflammation on the back; (**e**) Nummular-form eczema changed to exudative inflammatory erythema after 1 week; (**f**) Prurigo-forming papules and nodules on the upper extremities and trunk.

**Figure 5:**
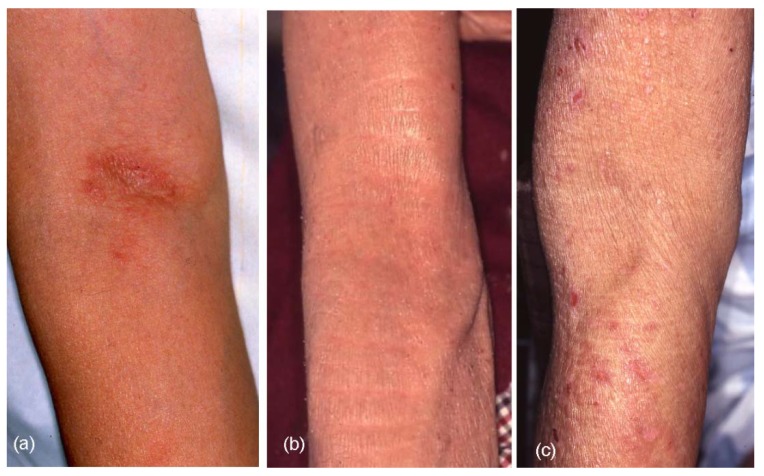
Lichenification (lichenified eczema) in the antecubital areas of elderly patients with atopic eczema (AE). (**a**) Localized lichenified eczema in the elbow fold; (**b**) Diffuse lichenified eczema in the elbow fold and flexure site of the arm; (**c**) Lichenified eczema around the scarcely involved elbow fold (reverse sign).

The results of clinical-picture analyses are summarized in Table 3. Although various forms of skin manifestations were observed in elderly patients with AE (Figure 4a–f), no significant difference was observed in the three types of elderly AE. On aggregate, eczematous dermatitis presented in 65.0% of cases involving the face and neck, 91.7% involving the trunk, 95.0% involving the upper extremities, and 83.3% involving the lower extremities in elderly AE patients. Hertoghe’s sign was recognized in 30% of elderly AE patients. Prurigo-forming papules and/or nodules on the trunk and extremities were observed in approximately 27% of patients. Exudative inflammatory erythema on the trunk and eczematous erythroderma were seen in 21.7% and 23.3% of patients, respectively. In regard to lichenified eczema (lichenification) in the extremities, lichenification was observed in 73.3% of extensor sites of the upper extremities and/or wrist, and 51.7% of extensor sites of the lower extremities. Lichenification in the elbow and knee folds showed relatively low positive rates as 23.3% (localized form, 10.0%; diffuse form, 13.3%) of the elbow folds and 18.3% (localized form, 8.3%; diffuse form, 10.0%) of the knee folds. Although 76.7% and 81.7% of elderly AE patients showed no lichenification in the elbow or knee folds, respectively, the reverse sign of lichenification around the folds was observed in 40.0% of patients in antecubital areas and 11.7% of patients in popliteal areas. Furthermore, 10% (IgE-allergic AE, 2.6%; non-IgE-allergic AE, 38.5%) of elderly AE patients reported a history of lichenified chronic eczema in the elbow and knee folds when they lacked lichenification in those folds at the time of medical examination. Lichenification in the antecubital areas (Figure 5a–c) in the three types of the elderly AE patients was as follows: localized, 5.3%; diffuse, 18.4%; around the folds, 39.5% in IgE-allergic AE, localized, 11.1%; diffuse,11.1%; around the folds, 33.3% in indeterminate-allergic AE, and localized, 23.1%; diffuse, none; and around the folds, 46.2% in non-IgE-allergic AE.

**Table 3:** Skin manifestations in elderly patients with atopic eczema (AE).

Skin Manifestations ¶	IgE-allergic AE (*n* = 38) *n* (%)	Indeterminate-Allergic AE (*n* = 9) *n* (%)	Non-IgE-Allergic AE (*n* = 13) *n* (%)	Total (*n* = 60) *n* (%)
Face and neck				
Eczema	26 (68.4)	4 (44.4)	9 (69.2)	39 (65.0)
Hertoghe’s sign	12 (31.6)	1 (11.1)	5 (55.6)	18 (30)
Trunk				
Eczema	36 (94.7)	8 (88.9)	11 (84.6)	55 (91.7)
Prurigo	11 (28.9)	2 (22.2)	3 (23.1)	16 (26.7)
Exudative inflamm.	10 (26.3)	2 (22.2)	1 (7.7)	13 (21.7)
Elbow folds				
Lichenification				
Localized	2 (5.3)	1 (11.1)	3 (23.1)	6 (10.0)
Diffuse	7 (18.4)	1 (11.1)	0 (-)	8 (13.3)
None	29 (76.3)	7 (77.8)	10 (76.9)	46 (76.7)
Around the folds	15 (39.5)	3 (33.3)	6 (46.2)	24 (40.0)
Upper extremities				
Lichenification	30 (78.9)	6 (66.7)	8 (61.5)	44 (73.3)
Eczema	37 (97.4)	9 (100)	11 (84.6)	57 (95.0)
Prurigo	13 (34.2)	2 (22.2)	2 (15.4)	17 (28.3)
Knee folds				
Lichenification				
Localized	4 (10.5)	0 (-)	1 (7.7)	5 (8.3)
Diffuse	6 (15.8)	0 (-)	0 (-)	6 (10.0)
None	28 (71.8)	9 (100)	12 (92.3)	49 (81.7)
Around the folds	5 (12.8)	0 (-)	2 (15.4)	7 (11.7)
Lower extremities				
Lichenification	22 (57.9)	2 (22.2)	7 (53.8)	31 (51.7)
Eczema	32 (84.2)	7 (77.8)	12 (92.3)	50 (83.3)
Prurigo	11 (28.9)	2 (22.2)	3 (23.1)	16 (26.7)
Erythroderma	10 (26.3)	1 (11.1)	3 (23.1)	14 (23.3)

¶ Eczema, mainly chronic eczematous dermatitis; Hertoghe’s sign, loss of lateral eyebrows; Prurigo, prurigo forming papules and/or nodules; Exudative inflamm., exudative inflammatory erythema; Localized, localized lichenification in the folds; Diffuse, diffuse lichenification in the flexure sites of upper or lower extremities including the folds; None, lichenification were not observed in the folds; Around the folds, lichenification around the unaffected folds of elbows and knees in the flexure sites of the extremities; Upper extremities, eczema and/or prurigo in both sites of upper extremities and lichenification in extensor sites of upper extremities and/or wrists; Lower extremities, eczema and/or prurigo in both sites of lower extremities and lichenification in the extensor sites of lower extremities.

### 3.2. Treatments and Outcomes

**Figure 6:**
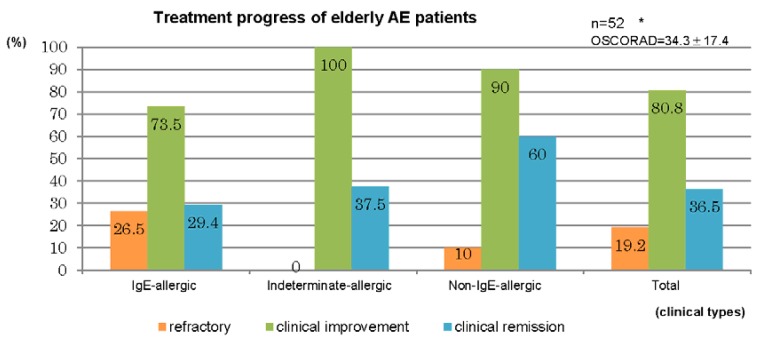

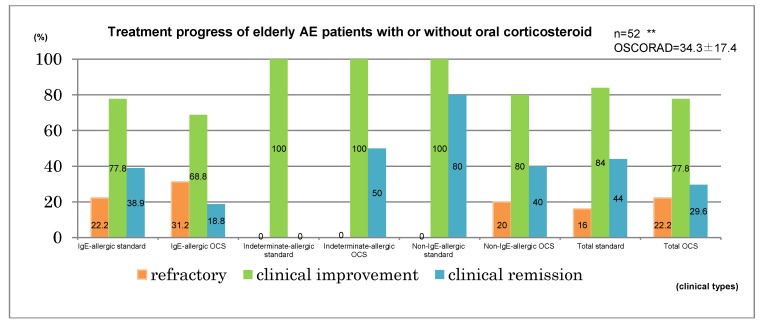
Treatment progress of elderly patients with atopic eczema. * IgE-allergic: *n* = 34, OSCORAD = 38.5 ± 17.6; Indeterminate-allergic: *n* = 8, OSCORAD = 24.1 ± 7.8; Non-IgE-allergic: *n* = 10, OSCORAD = 28.5 ± 18.2. ** IgE-allergic standard: *n* = 18, OSCORAD = 35.7 ± 16.3; IgE-allergic OCS: *n* = 16, OSCORAD = 41.6 ± 19.1; Indeterminate-allergic standard: *n* = 2, OSCORAD = 28.7 ± 6.4; Indeterminate-allergic OCS: *n* = 6, OSCORAD = 22.5 ± 8.0; Non-IgE-allergic standard: *n* = 5, OSCORAD = 19.3 ± 7.9; Non-IgE-allergic OCS: *n* = 5, OSCORAD = 37.7 ± 21.7; Total standard: *n* = 25, OSCORAD= 31.8 ± 15.6; Total OCS: *n* = 27, OSCORAD = 36. 7± 18.9. Abbreviations: AE, atopic eczema; standard, standard treatments (including topical corticosteroids and moisturizer, oral antihistamines, oral anti-allergic drugs, and topical tacrolimus); OCS, standard treatment with oral corticosteroids; OSCORAD, Objective Severity Scoring of Atopic Dermatitis (mean ± SD).

Therapeutic results for the 52 cases in which treatment of AE was performed in our department for a duration of ≥6 months are summarized in Figure 6. Treatment was performed by the dermatologists in accordance with the atopic dermatitis guidelines of the Japanese Dermatological Association [12]. A clinical improvement was observed in elderly cases comprising 73.5% of IgE-allergic AE, 100% of indeterminate-allergic AE, 90% of non-IgE-allergic AE, and 80.8% of the total. Clinical remission was obtained in elderly cases for 29.4% of IgE-allergic AE, 37.5% of indeterminate-allergic AE, 60% of non-IgE-allergic AE, and 36.5% of the total (Figure 6, upper bar chart). Standard treatments, which included topical corticosteroid and moisturizer, topical tacrolimus, oral antihistamines, and oral anti-allergic drugs [1,12], had been applied for 25 cases. By the standard treatments, clinical improvement was observed in the elderly cases of 77.8% of IgE-allergic AE, 100% of indeterminate-allergic AE, 100% of non-IgE-allergic AE, and 84% of the total, and clinical remission was obtained in elderly cases of 38.9% of IgE-allergic AE, 0% of indeterminate-allergic AE, 80% of non-IgE-allergic AE, and 44% of the total. Oral corticosteroid therapy with the standard treatments had been carried out for 27 cases. In three of the 27 cases, the oral corticosteroid had originally been started as therapy for complicated BA. With oral corticosteroid and standard treatments, clinical improvement was observed in elderly cases of 68.8% of IgE-allergic AE, 100% of indeterminate-allergic AE, 80% of non-IgE-allergic AE, and 77.8% of the total, and clinical remission was obtained in the elderly cases of 18.8% of IgE-allergic AE, 50% of indeterminate-allergic AE, 40% of non-IgE-allergic AE, and 29.6% of the total (Figure 6, lower bar chart). Mean initial dosage of oral corticosteroid was 7.7 ± 4.1 mg/day (IgE-allergic AE, 8.6 ± 4.7 mg/day; indeterminate-allergic AE, 5.0 ± 3.1 mg/day; non-IgE-allergic AE, 7.5 ± 1.8 mg/day) in prednisolone-equivalents. As other supplementary treatments, oral cyclosporine therapy (100 mg/day) and narrowband ultraviolet b (UVB) therapy were performed in two cases (IgE-allergic AE, *n* = 1; indeterminate-allergic AE, *n* = 1) and four cases (IgE-allergic AE only), respectively.

### 3.3. Complications and Final Prognosis

Coexisting/underlying disorders in the 60 cases of elderly AE were as follows. Incidence of hypertension was highest, at 53.3%, followed by heart disease (26.7%), spinal disease (25.0%), and cerebrovascular disease (21.7%). Complications of diabetes mellitus and chronic renal disease were observed at frequencies of 16.7% and 8.3%, respectively.

In cases of IgE-allergic AE, indeterminate-allergic AE and non-IgE-allergic AE, incidences were as follows: hypertension—58.3%, 44.4%, and 53.8%; heart disease—23.7%, 33.3%, and 30.8%; spinal disease—23.7%, 11.1%, and 38.5%; cerebrovascular disease—26.3%, 7.7%, and 22.2%; diabetes mellitus—18.4%, 22.2%, and 7.7%; and chronic renal disease—10.5%, 0%, and 7.7%, respectively. In each disorder, no significant differences in incidence were recognized among the three types of elderly AE. Steroid-induced diabetes mellitus with mild symptoms relevant to oral corticosteroid therapy was found in one patient with IgE-allergic AE and a low compliance to oral therapy.

**Figure 7:**
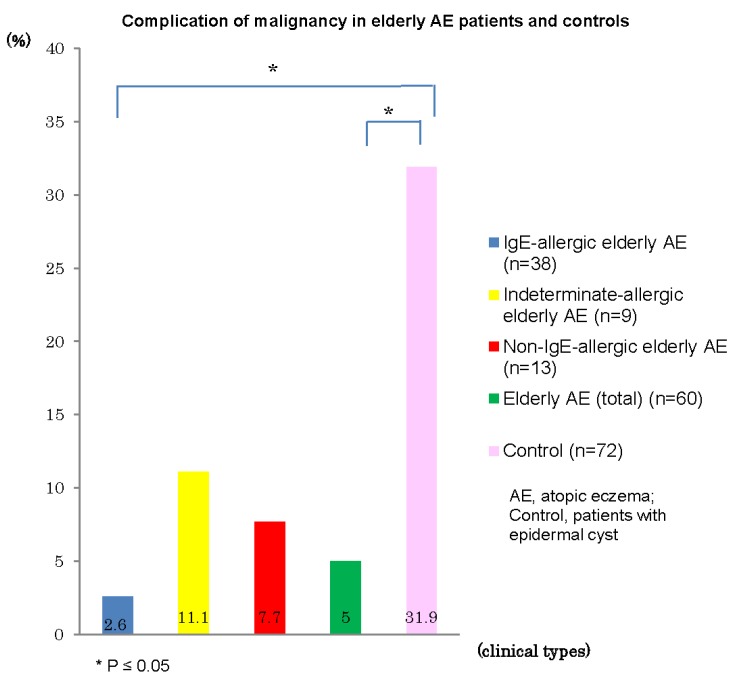
Complication of malignancy in elderly patients with atopic eczema and controls.

Complications of malignancy in personal histories and/or in coexisting/underlying disorders were recognized in three (5%) of the 60 elderly AE patients: rectal cancer and prostatic carcinoma in one patient with IgE-allergic AE, prostate carcinoma in one patient with indeterminate-allergic AE, and stomach cancer in one patient with non-IgE-allergic AE. The incidences of malignancy in elderly patients with IgE-allergic AE (2.6%) and with AE total (5%) were significantly lower than that (31.9%) of control patients with epidermal cysts (Figure 7). In control subjects, 23 (4 with double cancer) of the 72 patients showed complications of malignancy, as follows: pulmonary carcinoma, *n* = 5; stomach cancer, *n* = 4; colon cancer, *n* = 4; bladder cancer, *n* = 3; mammary carcinoma, *n* = 2; malignant lymphoma, *n* = 2; gallbladder and bile duct carcinoma, *n* = 2; basal cell carcinoma, *n* = 1; and other internal malignancy, *n* = 5. In addition, precancerous skin lesions, e.g., solar keratosis, were observed in two control subjects, but not in elderly AE patients.

The number of patients in whom death was confirmed by our hospital as of the end of 2014 was nine (IgE-allergic AE, *n* = 6; indeterminate-allergic AE, *n* = 1; non-IgE-allergic AE, *n* = 2). Mean age at death was 86.3 ± 6.8 years (IgE-allergic AE, 87.5 ± 7.9 years; indeterminate-allergic AE, 86 ± 0 years; and non-IgE-allergic AE, 83 ± 5.7 years). Causes of death were pneumonia, *n* = 3; respiratory failure, *n* = 1; fulminant hepatitis, *n* = 1; renal failure, *n* = 1; and natural death, *n* = 3. Among the identified patients, no deaths were attributable to malignancy. On the other hand, the mean age at death was 81.1 ± 4.2 years in the nine control subjects in whom death was confirmed by our hospital, and the cause of death for four of these nine subjects (44.4%) was malignancy. The incidence of death by malignancy among elderly patients with IgE-allergic AE (0%) and with AE total (0%) was lower than that (44.4%) in control subjects, but no significant difference was identified due to the small number of cases involved. In addition, most patients who died had been continuing treatment for AE until just before death.

## 4. Discussion

Although the limitations of retrospective analysis and small sample size must be considered, several characteristic of elderly AE became apparent from the present study of elderly patients with AE (*n* = 60) using clinical classifications of IgE-allergic AE (*n* = 38), indeterminate-allergic AE (*n* = 9) and non-IgE-allergic AE (*n* = 13) based on serological atopic diathesis.

From the analyses of clinical characteristics (Table 1), a male predominance and lower incidence of ichthyosis were observed in elderly patients with IgE-allergic AE. A previous study suggested a strong association between filaggrin mutation of the Ichthyosis-related gene and the development of AE in IgE-allergic AE patients [13], while a tendency toward a lower incidence of ichthyosis in IgE-allergic AE compared to non-IgE-allergic AE was reported in another study [14]. In regard to the onset of elderly AE (Figure 1, Table 2), two thirds of overall patients showed senile onset of AE symptoms. In elderly patients with IgE-allergic AE, onset of AE was observed in every phase of life, *i.e.*, early childhood, late childhood, adolescence, young adulthood, late adulthood, and senile phases. In elderly patients with non-IgE-allergic AE, onset of AE was seen in the early childhood, adolescence, late adulthood, and senile phases. Elderly patients with non-IgE-allergic AE showed a tendency toward female predominance for AE onset in the senile phase, although no statistically significant difference was evident. Meanwhile, in elderly patients with indeterminate-allergic AE, onset of AE was significantly concentrated in the senile phase with a male predominance. These results may suggest that these three types of elderly AE represent different phenotypes [15,16,17] not only in the serological characteristics but also in the clinical features. However, whether non-IgE-allergic elderly AE patients with early life (childhood, adolescence, or young adulthood) onset had continuously shown a non-IgE-allergic status remains unclear, and the possibility of mild symptom status shifting to IgE-allergic elderly AE could not be ruled out for either non-IgE-allergic elderly AE patients or indeterminate-allergic elderly AE patients [8,14].

Regarding the correlation between AE onset and clinical course, some interesting results became apparent (Table 2). In our previous analyses [3], three main patterns of onset were seen for elderly AE: senile onset; recurrence with a history of classic child AE; and recurrence and/or continuation of adult AE. In the present analyses, two patterns of AE onset and clinical course, *i.e.*, recurrent type with a history of outgrowing AE (outgrow-recurrence type) and continuous type, were confirmed both in cases of IgE-allergic elderly AE and non-IgE-allergic elderly AE with childhood onset and adolescence/young adulthood onset. Basically, recurrence of AE in the outgrow-recurrence type had arisen in the senile phase. When the fact that the present analyses were based on the memories of patients is taken into consideration, the continuous type might include cases of outgrow-recurrence type of infantile AE in which outgrowth had occurred in the early-childhood phase and recurrence happened in the adolescent/young adult phases [18].

In terms of the relationship with associated allergic diseases, some interesting findings were observed relevant to elderly AE and BA (Figure 2). In IgE-allergic elderly AE, elderly patients with continuous-type early life-onset AE showed a lower incidence of childhood BA than those with outgrow-recurrence type early life-onset AE. In addition, in elderly patients with continuous-type adolescence-/young adulthood-onset AE, asthmatic complications themselves had been uncommon. On the other hand, in elderly patients with senile onset of IgE-allergic AE, a complication of asthma was observed in every phase of life. Meanwhile, in indeterminate-allergic elderly AE and non-IgE-allergic elderly AE, a personal history of childhood BA was not observed in elderly patients. In general, childhood BA is well known to be a type of BA with an IgE-allergic predominance, frequently associated with childhood AE, and to carry an expectation of spontaneous remission (outgrowth) and subsequent recurrence [19,20]. The results in the present study suggest that the prognosis of AE at a younger stage of life may be predicted to some extent based on the incidence of childhood BA.

In analyses of serum-specific IgE for common environmental allergens among elderly patients with IgE-allergic AE (Figure 3), the positive rate of *Der f* peaked at 83.8%, followed by 67.6% for house dust and 56.8% for Japanese cedar, and mean positive scores also showed the highest values for *Der f* (2.65), Japanese cedar (2.38), house dust (2.2), and cat dander (2.2). Compared with our previous results [3], in which comparatively severe cases of elderly AE were examined, positive rates of food allergens were relatively lower in the present analysis. These findings may suggest that airborne allergens are more common and take precedence over food allergens in terms of associations with allergic sensitization in elderly patients with IgE-allergic AE.

In the analysis of skin manifestations (Table 3), eczematous dermatitis in elderly patients with AE was observed widely over the face and neck, trunk and extremities, as for AE in the childhood, adolescence, and young adulthood phases. However, similar to the results of our previous report [3], lichenification in the elbow and knee folds is a characteristic feature of classic AE in the early life phases, but was observed only in small numbers of elderly patients with AE. In elderly patients with IgE-allergic AE, localized and diffuse lichenification in the elbow folds as the easiest parts of physical examination were observed in 5.3% and 18.4% of patients, respectively. Absence of lichenification at the elbow folds was seen in 76.3% of patients. However, the reverse sign of lichenification around the folds was found in 39.5% of patients. Flexural lichenification in the antecubital areas was thus observed in 63.2% of elderly patients with IgE-allergic AE (Figure 5). Eczematous erythroderma was seen in 23.3% of elderly patients with AE. In general, clinical diagnosis of erythrodermic AE should be conducted carefully, since patients with senile erythroderma due to causes other than AE could also show high serum levels of total IgE and thymus activation-regulated chemokine (TARC) [21].

From the analyses of treatments and outcomes (Figure 6), it became apparent that clinical improvement and clinical remission were observed in 80.8% and 36.5% of cases with elderly AE, respectively. Compared to cases of the other two types, cases of IgE-allergic AE were relatively intractable. For severe cases and/or elderly cases of with a lower ability to use topical treatments, oral corticosteroid therapy with standard treatment had been carried out; clinical improvement was observed in 77.8% (IgE-allergic AE, 68.8%; indeterminate-allergic AE,100%; and non-IgE-allergic AE, 80%) of cases, and clinical remission was obtained in 29.6% (IgE-allergic AE, 18.8%; indeterminate-allergic AE, 50%; and non-IgE-allergic AE; 40%) of cases. These results indicate that even in intractable cases of IgE-allergic elderly AE, oral corticosteroid therapy could reduce the dosage of prednisolone to ≤5 mg/day (daily or occasional use) in approximately 70% of cases and be withdrawn in approximately 20% of cases when used together with suitable standard treatments [1,12] and therapeutic patient education [22]. Steroid-induced diabetes mellitus associated with oral corticosteroid therapy was found in only one patient (6.3%) with IgE-allergic AE and low compliance to oral therapy.

In the analyses of complications and final prognosis, it became apparent that most elderly patients with AE have some complications of non-allergic coexisting/underlying disorders (e.g., hypertension, heart disease, cerebrovascular disease, and diabetes mellitus) that differ markedly from the characteristics of other age groups of AE onset. The incidence of complications did not show any marked divergence from trends for the incidence in the general population of elderly Japanese [23]. In practical medical examinations, however, the existence of these coexisting/underlying disorders brought about difficulties in differentiating elderly AE from other itchy conditions, and resulted in restrictions on treatment options, such as oral corticosteroids and cyclosporine. The present analysis demonstrated that the incidence of malignancy (2.6%) in elderly patients with IgE-allergic AE was significantly lower than that in control subjects (31.9%) (Figure 7). This result is supported by some data [24], but not by other results [25,26] in other age groups of AE reported previously. This discrepancy may originate in differences between age groups of patients, history of treatments, or ethnic backgrounds of patients in each study. However, elderly patients with IgE-allergic AE may be speculated to have some immunological peculiarity acting against malignancy when taking into consideration findings such as the IgE-allergic reaction representing a kind of over-response against foreign substances, and the various types of immune responses other than immediate-type IgE-allergy to exclude foreign substances revealed in older patients with IgE-allergic AE [27]. This hypothesis may be supported by analysis of the incidence of mortality from malignancy, in which elderly patients with IgE-allergic AE tended to show a lower incidence (0%) than control subjects (44.4%). However, no significant difference was identified since the number of cases was small. In addition, the chief cause of death among elderly Japanese (65–89 years old) is malignancy, followed by heart disease and pneumonia [23].

These findings may bring some comfort to patients, and provide important supplementary information for prospective therapy with immunomodulatory agents, biological preparations, and immunotherapy [15,16,28] for the treatment of elderly AE. In addition, the majority of patients who died had been continuing treatment for AE until just before death. Most elderly AE patients thus could be considered to reach the end of life with AE.

## 5. Conclusions

AE in the elderly is a new subgroup of AE. AE can clearly become an allergic disease lasting a lifetime [29]. Elderly patients with AE showed some specific clinical characteristics, such as a male predominance, existence of several patterns of onset and clinical course, associations with IgE allergic status and asthmatic complications, and a lower incidence of lichenified eczema at the elbow and knee folds. In terms of treatments and outcomes, powerful anti-inflammatory treatments like oral corticosteroids are needed in some cases of elderly AE. In terms of complications and final prognosis, most elderly AE patients reached the end of life with AE, but patients with IgE-allergic AE showed a significantly lower incidence of malignant complications and an intriguing lack of death by malignancy. Further investigation of a larger cohort of elderly patients with AE is warranted.
